# Spatial Distribution of Inhibitory Innervations of Excitatory Pyramidal Cells by Major Interneuron Subtypes in the Auditory Cortex

**DOI:** 10.3390/bioengineering10050547

**Published:** 2023-05-01

**Authors:** Wen Zhong, Wenhong Zheng, Xuying Ji

**Affiliations:** 1 School of Traditional Chinese Medicine, Southern Medical University, Guangzhou 510515, China; zhong1981@smu.edu.cn; 2 Department of Physiology, School of Basic Medical Sciences, Key Laboratory of Psychiatric Disorders of Guangdong Province, Guangdong-Hong Kong-Macao Greater Bay Area Center for Brain Science and Brain-Inspired Intelligence, Key Laboratory of Mental Health of the Ministry of Education, Southern Medical University, Guangzhou 510515, China

**Keywords:** auditory cortex, spatial inhibitory priority, mental disorder, GABAergic interneurons, pyramidal neuron

## Abstract

Mental disorders, characterized by the National Institute of Mental Health as disruptions in neural circuitry, currently account for 13% of the global incidence of such disorders. An increasing number of studies suggest that imbalances between excitatory and inhibitory neurons in neural networks may be a crucial mechanism underlying mental disorders. However, the spatial distribution of inhibitory interneurons in the auditory cortex (ACx) and their relationship with excitatory pyramidal cells (PCs) remain elusive. In this study, we employed a combination of optogenetics, transgenic mice, and patch-clamp recording on brain slices to investigate the microcircuit characteristics of different interneurons (PV, SOM, and VIP) and the spatial pattern of inhibitory inhibition across layers 2/3 to 6 in the ACx. Our findings revealed that PV interneurons provide the strongest and most localized inhibition with no cross-layer innervation or layer specificity. Conversely, SOM and VIP interneurons weakly regulate PC activity over a broader range, exhibiting distinct spatial inhibitory preferences. Specifically, SOM inhibitions are preferentially found in deep infragranular layers, while VIP inhibitions predominantly occur in upper supragranular layers. PV inhibitions are evenly distributed across all layers. These results suggest that the input from inhibitory interneurons to PCs manifests in unique ways, ensuring that both strong and weak inhibitory inputs are evenly dispersed throughout the ACx, thereby maintaining a dynamic excitation–inhibition balance. Our findings contribute to understanding the spatial inhibitory characteristics of PCs and inhibitory interneurons in the ACx at the circuit level, which holds significant clinical implications for identifying and targeting abnormal circuits in auditory system diseases.

## 1. Introduction

In the past decade, a global decline in the prevalence of numerous chronic diseases has been observed. However, mental disorders have exhibited a contrasting trend, emerging as the predominant burden on families and societies worldwide. The ambiguous pathological mechanisms underlying many mental illnesses often render treatments ineffective, emphasizing the urgency to investigate the root causes of these conditions. An increasing body of research suggests that the pathogenesis of several mental disorders—including epilepsy, schizophrenia, depression, and autism spectrum disorder—may be closely associated with an imbalance in excitation and inhibition within the brain.

This excitation–inhibition imbalance has been implicated in abnormal neural circuitry within the central auditory pathway, which has been linked to various forms of hearing loss. For instance, auditory verbal hallucinations (AVHs) have been connected to hyperactivity in the auditory cortex, as demonstrated by functional imaging studies. This aberrant disinhibition of the auditory cortex culminates in self-generated speech, resembling the experience of AVH episodes [1]. In the context of tinnitus, a plethora of research has pinpointed hyperexcitability in the auditory cortex as a critical factor contributing to its manifestation [2,3,4]. In vivo studies have revealed that blocking GABA inhibition solely in the auditory cortex can induce tinnitus [5], and salicylate-induced tinnitus can augment the spontaneous discharge of 70% of cells [6]. This elevated excitatory postsynaptic response has been observed in cases of acquired deafness, congenital deafness, and tinnitus as well [7].

These discoveries highlight the significance of maintaining equilibrium between excitation and inhibition for the precise encoding of intricate information. Both overexcitability and diminished inhibition can contribute to atypical cortical network activity, leading to the emergence of brain disorders. In this study, we utilized a multidisciplinary approach, incorporating optogenetics, transgenic mice, and patch-clamp recording on brain slices, to examine the microcircuit characteristics of distinct interneurons (PV, SOM, and VIP) and their inhibitory input to PCs across layers 2/3 to 6 in the ACx. Our study enhances the understanding of the spatial inhibitory characteristics between PCs and inhibitory interneurons in the ACx at the circuit level, offering significant clinical insights for the identification and targeted intervention of aberrant circuits in auditory system diseases. In the neural circuits of the cerebral cortex, although excitatory pyramidal neurons (PC) are the main components, the inhibition mediated by GABAergic neurons plays a more important role in the stability of the neural circuits. In the neocortex, the vast majority of inhibitory neurons are PV (parvalbumin)-, SOM (somatostatin)- and VIP (vasoactive intestinal peptide)-expressing neurons, which account for nearly 100% of neocortical GABAergic neurons [8,9]. PV neurons are responsible for feed-forward inhibition [10], gamma oscillation [11], excitation/inhibition balance [12] and modulation of the activity code of excitatory neurons [13]. PV interneurons-mediated changes in inhibitory neural circuits are thought to be involved in a variety of central system diseases and cognitive disorders, such as depression, anxiety and psychosis, as well as impaired learning ability and social behavior. Studies have shown that the functional silencing or reduced expression of PV interneurons in the somatosensory cortex is sufficient to generate seizures [14] and autism spectrum disorder (ASD) [15,16]. The activation of PV can antagonize pro-epileptic chemical pentylenetetrazol (PTZ)-induced seizures, so PV-PC inhibition is considered as a potential therapeutic target for anti-absence seizure therapy. SOM neurons regulate the state of cortical activity by complementing PV neurons and governing disynaptic inhibition [17]. Many pathological abnormalities are associated with changes in SOM interneurons. In the hippocampus, for example, a reduction in the amount of SOM is considered a marker of epilepsy, but the mRNA levels increase for SOM interneurons in the kainic acid (KA) model of temporal lobe epilepsy, suggesting that the seizures may trigger SOM expression, and their receptors may be targets for anticonvulsive drug therapy [18]. VIP interneurons have direct excitatory effects on PC, and they can also regulate the synaptic transmission and synaptic plasticity of PC dendrites through inhibitory effects [19]. Nowadays, VIP is thought to be an important modulator of synaptic transmission, network excitability as well as of learning and memory processes, and it has been associated with cognitive deficits in several central nervous system (CNS) diseases. In both patients of mesial temporal lobe epilepsy with hippocampal sclerosis (MTLE-HS) and animal models, VIP receptors are up-regulated, which has different effects on cognitive function of the brain through the activation of VPAC1 or VPAC2 receptors [20]. It has been confirmed that VIP interneurons play an important role in the pathology and treatment of neurological disorders, such as Alzheimer’s disease, Parkinsonism and ASD. These studies suggest that PV, SOM, and VIP interneurons are all involved in the cortical excitation–inhibition balance in their own way. Therefore, it is necessary to study the inhibitory neural circuit characteristics of these three inhibitory interneurons, which not only helps to understand the neural structure basis of normal brain function, but more importantly, the research from the level of neural circuit has important clinical translational significance for the pathogenesis of many diseases and the discovery of therapeutic targets.

The mammalian sensory cortex is typically divided into six layers, meaning that the type and function of neurons in each layer may be different. In different layers of the primary auditory cortex (A1), PV, SOM and VIP interneurons have completely different distribution characteristics, and their different connections with PC result in different auditory responses and behaviors [21,22,23]. It has been found that the neurons in A1 of mice with hearing loss have layer specificity, and the degree of inhibitory interneurons reduction in different layers is inconsistent. Moreover, the functional activities of the ACx column are unevenly reduced, such as noise deafness, presbycusis deafness and congenital deafness. These studies have shown that inhibitory neurons in different layers may be involved in different neural circuits to exert precise regional control over the processing of auditory information. Therefore, it is quite important to study the spatial distribution characteristics between different inhibitory interneurons and PC. At present, most of these studies focus on the somatosensory cortex, while studies on ACx are limited. Due to the structural differences between cortical regions, different cortical regions may have a variety of microcircuits [24], and the properties of known local circuits may not be used as a template for all cortical regions [25], so it is necessary to study the auditory cortex. In the present study, we used optogenetics and transgenic mice combined with whole cell recordings on brain slices. By analyzing the inhibitory efficiency (including inhibitory strength and range) and spatial inhibitory priority, we found that the inhibitory input provided by different types of inhibitory neurons had obvious regional distribution in the whole ACx. They work in different ways to maintain the normal network activity of the auditory center.

## 2. Materials and Methods

### 2.1. Animal Preparation

All experimental procedures used in this study were approved by the Animal Care and Use Committee of Southern Medical University, *Guangzhou, China*. Transgenic mouse lines aged 7–11 weeks (obtained from The Jackson Laboratory, Bar Harbor, ME, USA) used in this study were *C57BL/6-Tg(pv-cre)Smoc* (PV-Cre), *Ssttm2.1(cre)Zjh/J* (SOM-Cre), and *B6J.Cg-Viptm1(cre)Zjh/AreckJ* (VIP-Cre) [26]. To visualize and label the inhibitory neurons of desired types, PV-Cre, SOM-Cre, and VIP-Cre mice were crossed with the *B6.Cg-Gt(ROSA)26Sortm14(CAG-tdTomato)Hze/J* (Ai14, Cre-dependent tdTomato) reporter line.

### 2.2. Viral Injection

Viral injections were performed as previously described [27]. Adult transgenic mice were anesthetized with isoflurane (2% *v*/*v*), a small incision was made in the skin covering the ACx, and a craniotomy of a small hole (0.5 mm diameter) was drilled (temporal lobe, 2.2–3.64 mm caudal to the bregma). AAV2/9.EF1α. DIO. hChR2 (H134R). EYFP virus (Brain VTA, ~e^13^ virus particles per mL) was delivered to the ACx with a beveled glass micropipette of a tip size of 30–40 μm. For each injection, 40 nL of the viral solution was injected at a rate of 20 nL/min. We injected the virus into two locations (2.7 and 3.2 mm caudal to the bregma) and at two depths (300 and 600 μm). The pipette was allowed to rest for 7 min before withdrawal after each injection. Finally, we sutured the scalp and injected 0.1 mg/kg buprenorphine subcutaneously and returned the mice to the cage.

### 2.3. Slice Preparation

Slice recording was performed four weeks after viral injection. Cortical slices were prepared as our previous study [28,29]. After being anesthetized with isoflurane, the virus-injected mice were decapitated. The brains were quickly removed and immersed in cold (4 °C) dissecting solution (60 mM NaCl, 3 mM KCl, 1.25 mM NaH_2_PO_4_, 25 mM NaHCO_3_, 115 mM sucrose, 10 mM glucose, 7 mM MgCl_2_, 0.5 mM CaCl_2_; bubbled with 95% O_2_ and 5% CO_2_; pH = 7.4; 285–295 mOsm). A vibrating microtome (VT1000S, Leica, Shanghai, China) was used to cut 350 μm thick slices of the coronal cortex from the infected cerebral hemisphere, including the ACx area. The slice was transferred to the recording chamber at room temperature after 30 min of incubation in warm (35 °C) artificial cerebral spinal fluid (ACSF: 126 mM NaCl, 2.5 mM KCl, 1.25 mM NaH_2_PO_4_, 26 mM NaHCO_3_, 1 mM MgCl_2_, 2 mM CaCl_2_, 0.5 mM ascorbic acid, 2 mM sodium pyruvate, and 10 mM glucose, bubbled with 95% O_2_ and 5% CO_2_).

### 2.4. Electrophysiology

Electrophysiological recording was performed as previously described [30]. Recording was performed under an upright fluorescence microscope (BX51WI, Olympus, Tokyo, Japan) equipped with an infrared light source. Before each recording, the spatial expression of hChR2-EYFP fluorescence in each cortical slice was checked with a 4 × objective fluorescence microscope. In slices with good viral expression within the ACx and with an expression range of 800–1000 μm, we made whole-cell voltage clamp recordings on non-fluorescent excitatory cells of PV-ChR2, SOM-ChR2, and VIP-ChR2 slices by epifluorescence imaging in the ACx, respectively. EYFP-labeled inhibitory neurons were used for either specifically targeting or avoiding recording from those inhibitory neurons. During recording, TTX (tetrodotoxin, a sodium channel blocker, 1 μM) and 4-aminopyridine (a potassium channel blocker, 1 mM) were applied in the bath solution in all optogenetic experiments to obtain monosynaptic inhibitory responses [31]. For voltage-clamp recordings, we used a glass pipette (4–7 MOhm resistance) filled with a caesium-based internal solution (125 mM Cs-gluconate, 5 mM TEA-Cl, 4 mM MgATP, 0.3 mM GTP, 10 mM phosphocreatine, 10 mM HEPES, 1 mM EGTA, 2 mM CsCl, adjusted to pH 7.3 with CsOH). For current-clamp recordings, we used a potassium-based internal solution (125 mM K-gluconate, 10 mM HEPES, 10 mM EGTA, 4 mM Mg-ATP, 0.3 mM GTP, 2 mM KCl, 0.1 mM CaCl_2_, and 8 mM phosphocreatine sodium, adjusted to pH 7.3 with KOH). The pipette and whole-cell capacitances were completely compensated, and the initial series resistance was compensated for 50% at a 100 μs lag. Inhibitory postsynaptic currents (IPSCs) were recorded with the membrane potential voltage clamped at 0 mV. Signals were filtered at 2 kHz and sampled at 10 kHz.

### 2.5. Photostimulation

The photostimulation was controlled by an illuminator (Polygon 400, Mightex, Pleasanton, CA, USA), which delivered 470 nm blue light square of a 3 ms pulse duration to activate ChR2. A facula, which is set as a 30 × 30 μm blue square, was randomly illuminated around the recorded PC until the entire ACx area was covered (including the location of the patched cell). There is no gap among the facula. Before each experiment, we calibrated the blue illumination to ensure that the stimulus was at the target position. The light intensity measured at the focal plane (measured at the tip of the fiber) is 2.3 mW/mm^2^. For each stimulus, we repeated 10 trials and averaged the responses. The pia surface of the brain slice was set as 0 μm, stratifying the ACx based on our previous research (Ji et al., 2016 [28]).

### 2.6. Data Analysis

Only data with the virus in the target were analyzed. The response was considered to be evoked only if the response amplitude exceeded the average baseline level by 2 standard deviations (SDs) of the baseline fluctuations and if the probability of consistent responses after repetitive stimulation was greater than 50%.

For the analysis of the inhibitory strength and range, we took the patched cell as the center and divided all the stimulus blue squares into different areas according to the number of circles. For example, the first lap consists of all the closest blue squares around the patched cell. The one outside of the first lap is the second one, and so on. Lap 0 means the stimulus square is on the patched cell. The farthest range is the tenth lap (almost no reaction can be recorded here). For the statistics of the average amplitude, each IPSC amplitude was normalized from averages of multiple (at least three) IPSCs when the stimulus square was on the recorded cell.

Custom-made MATLAB programs (R2012b; MathWorks, Natick, MA, USA) were used to analyze the offline data. OriginPro 8.0 and Excel 2007 were used to calculate the values of relevant parameters. One-way ANOVA (LSD’s tests for multiple comparisons) was used to compare means, and a *p* value < 0.05 was considered as statistically significant.

### 2.7. Biocytin Staining

To study the morphological structure of neurons, we used biocytin in this experiment to label soma, dendrites and axons of neurons by fluorescent staining. During the whole-cell recordings, 0.1% biocytin (from Sigma, St. Louis, MO, USA) was added to the internal solution and stably recorded for at least 30 min. Then, the glass pipette was removed slowly from the recorded neuron to prevent leakage of the biocytin and to maintain the normal morphology of the neuron. Brain slices were immersed in 4% PFA and left at 4 °C overnight. Then, the slices were equilibrated at room temperature for 1 h and transferred to 1× PBS. We added 500 μL 0.3% Triton X-100 to each brain slice and incubated it for 2 h with shaking at room temperature. After rinsing with 1× PBS, each brain slice was immersed in 500 μL streptavidin-Cy3 (dilution ratio of 1:200) and incubated for 4 h in the dark at room temperature. After cover slipping, we used a confocal microscope to observe the morphology of the neurons.

## 3. Results

### 3.1. Intrinsic Spiking Properties and ChR2-EYFP Expression in the Three Inhibitory Cell Types

**Figure 1:**
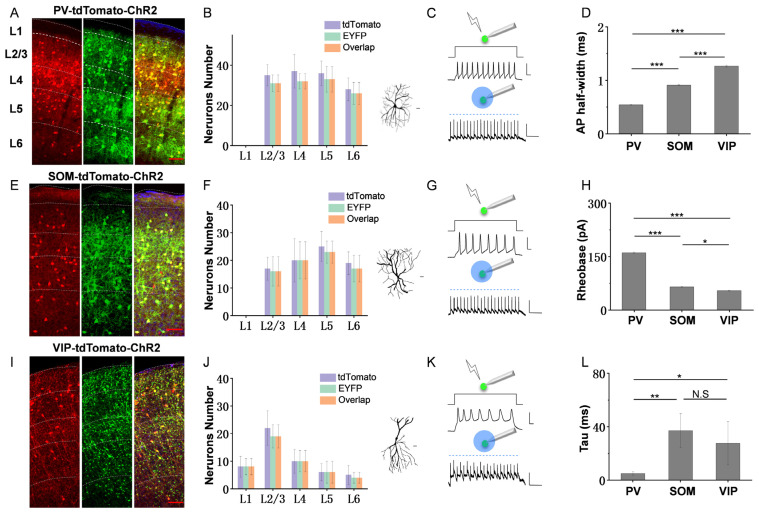
ChR2 expression and electrophysiological properties of different inhibitory neurons. (**A**,**E**,**I**) The example images showing the overlap between EYFP and the labeled inhibitory neurons (Cre-tdTomato line) of three subtypes of ACx slices, respectively. Red, labeled soma by Ai14. Green, ChR2 expressing. Orange, overlap expression of Ai14 and ChR2. Dashed curves, the boundary of different layers. (**B**,**F**,**J**) Left, the neurons number expressing tdTomato, EYFP and overlap in each layer, respectively. Right, reconstructed morphologies of the three interneurons. Scale bar, 20 μm. A total of 8 PV brain slices from 6 mice; 6 SOM brain slices from 6 mice; 7 VIP brain slices from 6 mice. (**C**,**G**,**K**) Top, action potentials (AP) of PV (**C**), SOM (**G**) and VIP (**K**) neurons by injecting 200 pA and 300 ms current in current-clamp. Scale bar, X: 50 ms, Y: 30 mV. Bottom, AP trains of PV (**C**), SOM (**G**) and VIP (**K**) neurons by activating ChR2 with a 10 Hz train of blue light. Scale bar, X: 50 ms, Y: 50 mV. Green circle, the recorded neurons expressing ChR2. Blue circle, the blue light for photo-stimualtion. (**D**) The comparison of AP half-peak width (±SD) across three subtypes of interneurons. (**H**) The comparison of AP rheobase (±SD) across three subtypes of interneurons. (**L**) The comparison of AP tau (±SD) across three subtypes of interneurons. A total of 15 PV neurons from 4 mice, 12 SOM neurons from 4 mice, and 10 VIP neurons from 3 mice. * *p* < 0.05, ** *p* < 0.01, *** *p* < 0.001, N.S, *p* > 0.05.

To confirm the infection efficiency of the virus, we made the quantification of virus-marker colocalization firstly. We injected an AAV vector encoding Cre-dependent hChR2 fused with EYFP into the ACx of the adult transgenic mouse lines PV-Cre-tdTomato, SOM-Cre-tdTomato and VIP-Cre-tdTomato [32] (see Section 2). After four weeks of viral expression, EYFP fluorescence-labeled interneurons expressed ChR2 through multiple layers of the ACx. The images in Figure 1A,E,I exhibited the tdTomato, hChR2-EYFP and overlap expression in the three subtypes of the brain slices, respectively. It is found that most inhibitory neurons have good viral expression within ACx. Then, we calculated the number of neurons expressing tdTomato, EYFP and the overlap in each layer, respectively. As shown in Figure 1B,F,J, most neurons expressing tdTomato had good overlap with EYFP in each layer of ACx. Those slices with poor spread of the virus and leaked expression on PCs were discarded to prevent interference with the experimental results. Since the L1 inhibitory neurons do not express PV and SOM markers [8], no labeled interneurons were observed in L1 of the PV and SOM slices. While VIP neurons were mainly distributed in the upper layer, there were few neurons in L5 and L6 [33]. The reconstructed morphologies showed that the PV neurons had denser local somato-dendrites and axonal arborizations compared with the others and exhibited the typical multipolar cell type (Figure 1B right panel). SOM neurons distinguished themselves by extending broad axonal arborization upward (Figure 1F right panel). VIP neurons mostly had a bipolar morphology with two main opposing vertically oriented dendrites and axonal arborizations into deep infragranular layers (Figure 1J right panel). These results are consistent with previous morphology studies of these inhibitory cell types [33,34,35,36], indicating the precise labeling of transgenic mice.

To re-confirm the labeled inhibitory cell type of transgenic mice and assure a high infection efficiency work of the virus, the basic spiking properties of the three inhibitory neurons were examined. Whole-cell recording in current-clamp mode was performed in fluorescence-labeled cells with a K^+^-based internal solution. PV neurons displayed the greatest number of action potentials (AP) [37], high-frequency and non-accommodating fast spiking responses to positive current injections (Figure 1C top panel), and they exhibited the shortest average AP width, which was measured at 50% of the peak amplitude (Figure 1D, 0.54 ± 0.05 ms for PV; 0.91 ± 0.13 ms for SOM; 1.26 ± 0.15 ms for VIP; mean (±SD)). In addition, they have the largest rheobase, which is the minimal current intensity to evoke an AP (Figure 1H, 160.38 ± 14.33 pA for PV; 65.00 ± 9.49 pA for SOM; 53.75 ± 8.35 pA for VIP; mean (±SD)). Meanwhile, SOM and VIP neurons exhibit adaptive spiking properties (Figure 1G,K top panels) with relatively lower spiking frequency and higher excitability, particularly for VIP. The membrane time constant (Tau) of PV neurons was the shortest (Figure 1L, 5.02 ± 1.30 ms for PV; 36.90 ± 12.78 ms for SOM; 27.61 ± 16.10 ms for VIP; mean (±SD)), which was obtained from the membrane potential change in an exponential fit by hyperpolarizing the current injection (−10 pA). These electrophysiological properties were in good agreement with the cell’s known intrinsic properties [35,38]. The statistics in Figure 1G,H,I were from 15 PV neurons, 12 SOM neurons, and 10 VIP neurons. By recordings these hChR2-EYFP expression interneurons, it is found that they all spiked reliably to blue LED pulses applied to the cortical slices (10 Hz), indicating the high work efficiency of the virus (Figure 1C,G,K bottom panels).

### 3.2. The Inhibitory Efficiency from Three Subtypes of Interneurons to PCs in Each Layer of ACx

**Figure 2:**
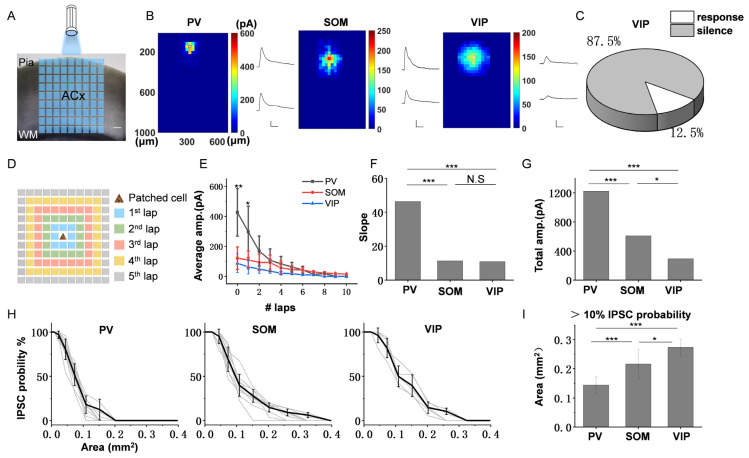
The inhibitory efficiency from three subtypes of interneurons to L2/3 PCs. (**A**) The illustration shows the ChR2-expressing interneurons activated by illumination on ACx slice. Blue square, 470 nm photostimulation with size of 30 × 30 μm. WM, white matter. Scale bar, 100 μm. (**B**) Left, three example color maps depict the IPSCs amplitudes and response range across three subtypes of interneurons, respectively. Each color square represents an IPSC (averages of at least three IPSCs). Right, two example traces of a labeled asterisk and the triangle location in color maps, and the asterisk also represents the soma locations of the recorded PCs. Scale bar for PV, X: 200 ms, Y: 300 pA. Scale bar for SOM and VIP, X: 200 ms, Y: 100 pA. (**C**) Pie chart showing that most responses are silent for the inhibition of VIP-PCs. (**D**) The schematic diagram exhibits that all the responses are assigned to different numbers of laps according to the location of the stimulus square. Each lap appears as a different colored square (for convenience, we only show five laps here; the actual data analysis includes all reactions within ten laps). (**E**) Summary of average amplitude in different laps (n = 10 for PV, 10 for SOM, 5 for VIP). Scale bar, 100 μm. (**F**) The slopes of the three curves in Figure (**E**). (**G**) Comparison of total amplitude within ten laps for three subtypes of interneurons. (**H**) IPSCs response probability in different stimulus area for three subtypes of interneurons. Gray lines, response probability of each recorded neuron. Black line, averaged response probability from gray lines. (**I**) Comparison of the areas corresponding to the response probability > 10% for three subtypes of interneurons. Scale bar, 100 μm. * *p* < 0.05, ** *p* < 0.01, *** *p* < 0.001, N.S, *p* > 0.05.

Here, we used the inhibitory strength and range to measure the inhibitory efficiency of different interneurons–PCs. To explore the inhibition in each layer of ACx, a 30 × 30 µm blue square was applied to randomly stimulate different areas of different transgenic ACx slices, and the IPSCs of the patched PCs were recorded until the entire ACx was stimulated (see methods, Figure 2A). The color map shows three example responses when recording L2/3 PCs (Figure 2B). We found that the responses from the PV slice were limited within a small area surrounding the recording PCs, whereas there existed a broader response area for SOM and VIP. However, the response amplitude showed an opposite result. The amplitudes of PV IPSCs were much bigger than those of SOM and VIP. It is worth noting that VIP neurons have less direct input to PCs; the low response probability is shown in Figure 2C (response, 5/40; silence, 35/40), whereas the response probabilities of PV and SOM are all 100%. It indicates that VIP neurons do not preferentially input to PCs, which is consistent with previous reports [39,40].

To compare the inhibitory strength of different interneurons–PCs, we divided the stimulus areas into 10 laps around the recording PCs (see methods, Figure 2D) and calculated the average amplitude of IPSCs of each lap, respectively (Figure 2E). The results showed that the amplitude of IPSCs was the largest when the stimulus position was on the recording cell. It decreased when the stimulus position moved farther from the recording cell, which is consistent in the three subtypes of interneurons. However, PV exhibited bigger IPSC amplitudes than the other two subtypes and then decayed rapidly; the average amplitude In the 2nd lap has dropped to close to SOM. On the contrary, SOM and VIP exhibited smaller IPSC amplitudes but decayed slower. By comparing the curve fitting of the three subtypes of interneurons (Figure 2F), we found that the slope of PV was significantly greater than that of SOM and VIP (−46.3 for PV, −10.91 for SOM, −11.33 for VIP), which suggests that PV has a strong and local inhibition, while SOM and VIP have a weak and extensive inhibition. To compare the total inhibitory strength of inhibitory input to L2/3 PCs across all layers for the three subtypes of interneurons, we calculated the sum amplitudes of all IPSCs within 10 laps (Figure 2G). The results showed that the strongest inhibition to L2/3 PCs was from PV neurons (PV: 1219.89 pA, SOM: 609.64 pA, VIP: 292.02 pA).

To compare the inhibitory range of interneurons–L2/3 PCs in each layer for the three subtypes of interneurons, we analyzed the probability of IPSCs by calculating the area of the rectangle corresponding to each stimulus lap, respectively. Figure 2H shows that all response probabilities decreased when the stimulus range expanded. To better observe the difference of inhibitory range, we calculated the area with more than 10% IPSCs probability (when less than 10% probability, most of the response disappeared), and we found that VIP neurons exhibited the most extensive inhibitory range, followed by SOM, and PV was the least (Figure 2I, 0.14 ± 0.03 mm^2^ for PV; 0.22 ± 0.05 mm^2^ for SOM; 0.27 ± 0.03 mm^2^ for VIP; mean (±SD)). This result suggests that different inhibitory neurons show diverse inhibitory efficiency to L2/3 PCs. PV neurons have the strongest inhibition, and VIP neurons have the widest inhibitory range.

**Figure 3:**
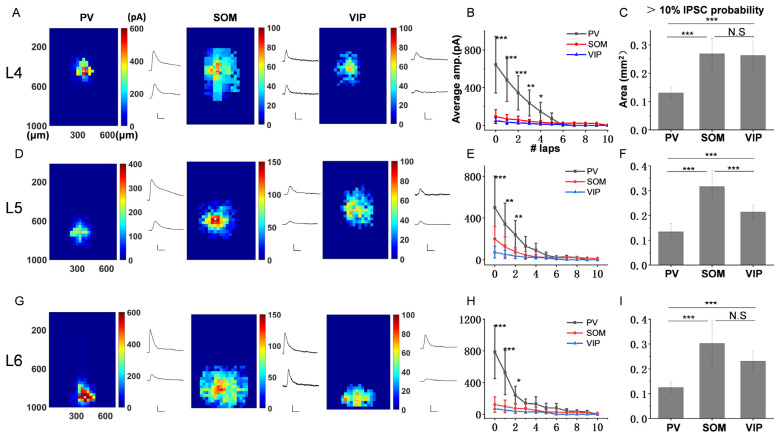
The inhibitory efficiency from three subtypes of interneurons to PCs across L4-L6. (**A**,**D**,**G**) Left, three example color maps showing the IPSCs amplitudes and response range across three subtypes of interneurons. Right, two example traces corresponding to the responses of the labeled asterisk and triangle location in color maps. Scale bar for PV, X: 200 ms, Y: 300 pA. Scale bar for SOM and VIP, X: 200 ms, Y: 100 pA. (**B**,**E**,**H**) Summary of average amplitude in different laps (L4: n = 8 for PV, 10 for SOM, 3 for VIP; L5: n = 9 for PV, 12 for SOM, 5 for VIP; L6: n = 10 for PV, 9 for SOM, 2 for VIP). (**C**,**F**,**I**) Comparison of the area corresponding to the response probability > 10% for three subtypes of interneurons. * *p* < 0.05, ** *p* < 0.01, *** *p* < 0.001, N.S, *p* > 0.05.

Following the same method, we explored the inhibitory efficiency of the three subtypes of interneurons to PCs in other layers. Figure 3A,D,G show the response color map and two IPSCs examples of L4, L5 and L6 PCs, respectively. Consistent with the results of L2/3 PC, the IPSCs provided by PV were much stronger than that of SOM and VIP. This can also be shown in the average amplitude graph of different laps (Figure 3B,E,H). By analyzing the slope of curve fitting, we found that PV decayed rapidly, while SOM and VIP decayed slower (Supplementary Figures S1A, S2A and S3A), which suggests that the inhibitory range of PV neurons is much smaller than that of SOM and VIP in all layers of ACx. The results of total IPSCs amplitude comparison showed that the strongest inhibitions to PCs in each layer were all from PV neurons (Supplementary Figures S1B, S2B and S3B), and there was little direct inhibition of VIP-PCs in each layer (Supplementary Figures S1C, S2C and S3C). These results indicated that the basic character of the inhibitory efficiency of three subtypes of interneurons to PCs is similar across all layers of ACx.

However, when we analyzed the area with more than 10% IPSCs probability, it is found that SOM and VIP neurons have the most extensive inhibitory range for L4 PCs (Figure 3C, 0.13 ± 0.02 mm^2^ for PV; 0.27 ± 0.05 mm^2^ for SOM; 0.26 ± 0.05 mm^2^ for VIP; mean (±SD)), SOM neurons have the most extensive inhibitory range for L5 (Figure 3F, 0.13 ± 0.03 mm^2^ for PV; 0.32 ± 0.06 mm^2^ for SOM; 0.21 ± 0.03 mm^2^ for VIP; mean (±SD)) and L6 PCs (Figure 3I, 0.13 ± 0.02 mm^2^ for PV; 0.30 ± 0.09 mm^2^ for SOM; 0.23 ± 0.04 mm^2^ for VIP; mean (±SD)). These results indicate that PV neurons supply the strongest and most local inhibition, while SOM and VIP neurons supply the weaker and wider inhibition, which is the common characteristic across all layers of ACx, but the inhibitory source of the broadest inhibitory range is different in each layer.

### 3.3. The Spatial Inhibitory Priority of the Three Subtypes of Interneurons in ACx

From the results of Figure 2I and Figure 3C,F,I, we also noticed that that SOM neurons had a wider inhibitory range for PCs in deep infragranular layers, including L5 and L6. Meanwhile, VIP neurons had a wider inhibitory range for upper supragranular PCs, such as L2/3. PV neurons have the narrowest inhibitory range in each layer. To further confirm the spatial inhibitory priority of different interneurons, we compared the following parameters of a single subtype of interneuron in different layers, including the total IPSCs probability, the area with more than 10% IPSCs probability, and the total response amplitude.

**Figure 4:**
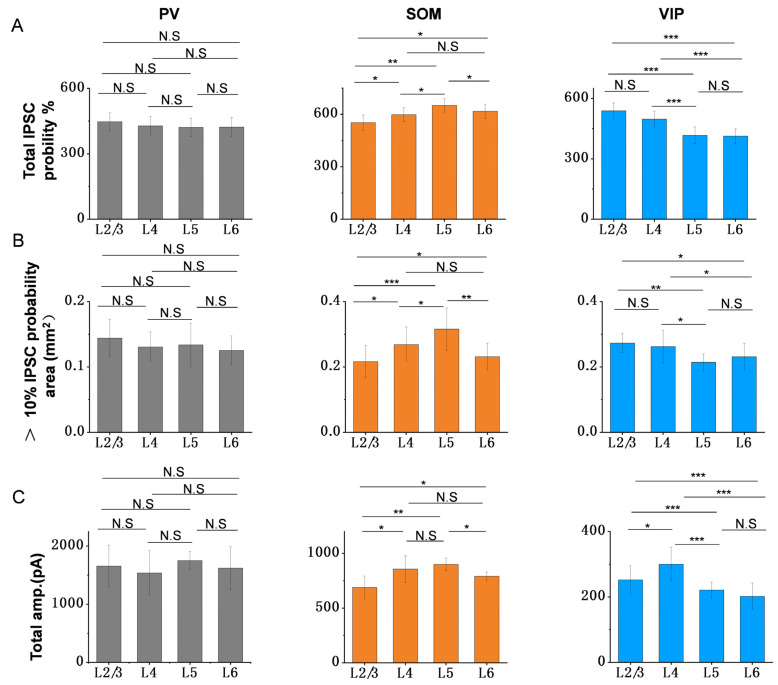
Spatial inhibitory priority among the three subtypes of interneurons. (**A**) Comparison of total response probabilities for different cell types in different layers. Gray column, data analysis of inhibition from PV to PCs. (**B**) Comparison of the area with more than 10% response probability for different cell types in different layers. Orange column, data analysis of inhibition from SOM to PCs. (**C**) Comparison of total amplitude for different cell types in different layers. Blue column, data analysis of inhibition from VIP to PCs. * *p* < 0.05, ** *p* < 0.01, *** *p* < 0.001, N.S, *p* > 0.05.

For the total IPSCs probability, we took the sum of the response probabilities within 10 laps and compared them among different layers. It is found that there is no significant difference among different layers for PV neurons (Figure 4A left panel, 447.11 ± 41.68 for L2/3; 428.48 ± 42.58 for L4; 421.45 ± 42.51 for L5; 422.61 ± 43.00 for L6; mean (±SD)). However, SOM and VIP neurons showed different results. The higher response probabilities of SOM neurons appeared in L4, L5 and L6, especially L5 (Figure 4A middle panel, 552.48 ± 43.25 for L2/3; 590.40 ± 40.59 for L4; 651.43 ± 38.55 for L5; 617.55 ± 41.07 for L6; mean (±SD)). The higher response probabilities of VIP neurons appeared in L2/3 and L4 (Figure 4A right panel, 539.39 ± 37.94 for L2/3; 497.66 ± 40 for L4; 417.07 ± 41.01 for L5; 412.71 ± 36.32 for L6; mean (±SD)). This is consistent with the results of the area with more than 10% IPSCs probability and total amplitude. SOM neurons have wider inhibitory range and bigger response amplitude in deep infragranular layers (L4, L5 and L6), especially L5 (Figure 4B,C middle panel, total amplitude: 688.76 ± 104.69 pA for L2/3; 857.19 ± 121.41 pA for L4; 900.42 ± 58.34 pA for L5; 790.55 ± 40.56 pA for L6; mean (±SD); the data of the area with more than 10% IPSCs probability are shown in Figure 2 and Figure 3). On the contrary, VIP neurons exhibited a wider inhibitory range and bigger response amplitude in the upper supragranular layer, including L2/3 and L4 (Figure 4B,C right panel, total amplitude: 252.07 ± 43.46 pA for L2/3; 300.11 ± 52.08 pA for L4; 221.01 ± 24.50 pA for L5; 202.02 ± 40.23 pA for L6; mean (±SD); the data of the area with more than 10% IPSCs probability are shown in Figure 2 and Figure 3). Meanwhile, PV neurons showed no spatial inhibitory priority (Figure 4B,C left panel, total amplitude: 1655.81 ± 358.98 pA for L2/3; 1537.70 ± 382.35 pA for L4; 1750.12 ± 156.11 pA for L5; 1619.84 ± 367.59 pA for L6; mean (±SD); the data of the area with more than 10% IPSCs probability is shown in Figure 2 and Figure 3). These results indicate that the spatial inhibition from different inhibitory neurons to PCs does have a varied regional priority, and different cell types may have their own priority region to work when processing information in the intracortical projection of ACx.

### 3.4. Summary of Inhibitory Efficiency and Spatial Inhibitory Priority of Different Interneurons to PCs

**Figure 5:**
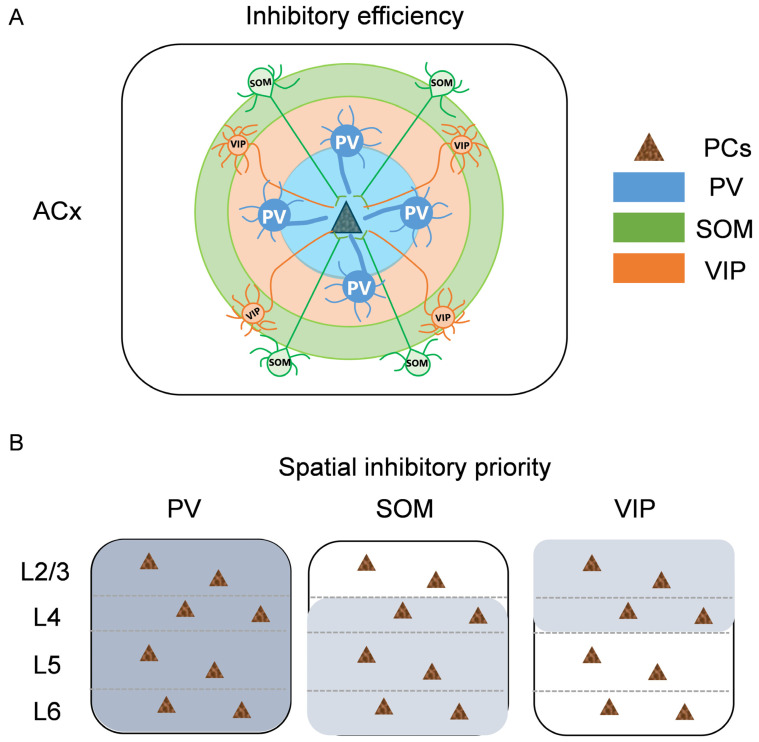
Summary diagram of microcircuit characteristics for different subtypes of inhibitory neurons. (**A**) Inhibitory efficiency of different interneurons–PCs. Triangle, PCs. Blue, PV. Green, SOM. Orange, VIP. Circle, the inhibitory range of the interneurons to PCs; the bigger the circle means the wider the range, the thicker the line means the stronger the inhibitory input. (**B**) Inhibitory spatial priority of different interneurons–PCs. Gray, the priority inhibitory region. The darker the gray, the stronger the inhibition.

The above results show that the three subtypes of inhibitory neurons in ACx have their own special microcircuits characteristics in terms of inhibitory efficiency and spatial inhibitory priority. As shown in Figure 5, for inhibitory efficiency, PV neurons provide the strongest inhibitory input to adjacent PCs within a local range, while SOM and VIP neurons provide weaker inhibitory input to PCs in a wider range. These characteristics are consistent across all layers of ACx, without layer specificity, but the inhibitory source of the broadest innervation range is different in each layer, either SOM or VIP neurons.

For spatial inhibitory priority, the three subtypes of interneurons exhibited distinct characteristics. Due to the local inhibitory range of PV neurons, most of the response areas were focused within the same layer, with almost no cross-layer innervation, and the strong inhibitory input was distributed evenly throughout the ACx. However, SOM and VIP neurons showed obvious spatial inhibitory priority. SOM neurons were more likely to inhibit PCs in deep infragranular layers, while VIP neurons preferred to inhibit PCs in upper supragranular layers. In this manner, the weak inhibitory input is evenly distributed throughout the ACx.

Therefore, we believe that when auditory information is processed in ACx, PV neurons participate in the role of strong and precise tuning in local circuits as the source of the strongest inhibitory input, which is uniformly distributed in each layer of ACx. On the contrary, by providing weak inhibitory input, SOM and VIP neurons can fine-tune information in wider areas. In this manner, the inhibitory input to PCs is spatially balanced throughout the ACx. The three subtypes of interneurons play distinct roles in the local circuits and information processing of ACx through their own special working patterns.

## 4. Discussion

In this study, we investigated the microcircuit characteristics of neural circuits between various inhibitory interneurons and excitatory neurons across layers 2/3 to 6 in the mouse auditory cortex (ACx). We employed whole-cell recordings from pyramidal cells (PCs) in transgenic mice expressing green fluorescent protein in specific inhibitory neuron populations combined with optogenetics. As layer 1 contains few PCs, it was not included in our study.

Our findings demonstrate that parvalbumin (PV) neurons, the most potent inhibitory input source for all layers of PCs, provide the strongest inhibition to adjacent PCs among the three interneuron subtypes. The inhibitory range of PV-PCs is localized, inhibiting PCs within the same or adjacent layers without cross-laminar inhibition. These characteristics are consistent with other brain regions and are similar across all layers of the ACx. PV neurons have no distinct spatial inhibitory preferences, ensuring an evenly distributed inhibition in each layer. PV neurons thus exert precise regulation to each layer of PCs evenly, with strong and local inhibition and no layer specificity throughout the ACx. This is crucial for the processing of fine sounds in the ACx, enabling the rapid and precise processing of auditory information and discrimination of new auditory stimuli. The reduced expression or functional abnormalities in PV neurons may lead to deficits in rapid auditory processing, such as autism spectrum disorder (ASD) [7], generalized spike-wave discharges on electroencephalograms, and other diseases [41,42]. In contrast to PV, somatostatin (SOM) neurons exhibit relatively weak inhibitory input with a broader inhibitory range. SOM neurons display spatial inhibitory preferences, with larger inhibitory postsynaptic currents and response probabilities found in deep infragranular layers, particularly layer 5 [21,43,44]. SOM neurons exert fine-scale regulation to deep-layer PCs with weak and extensive input, without layer specificity throughout the ACx. The abnormal function of SOM neurons could lead to excitotoxicity and the death of excitatory neurons, resulting in hearing disorders, spatial learning defects, and memory decline. In particular, when the SOM neuron circuit is abnormal in the deep layer of the cortex, it will also cause spatial learning defects and memory decline, such as the decrease in SOM expression in the cortex caused by old age. SOM appears to have a potential neuroprotective effect in preventing epileptic activity [45,46] and is involved in learning and memory retention [47,48].

Similar to SOM, vasoactive intestinal peptide (VIP) neurons also exhibit weak inhibitory input and a wide range. Unlike the other two interneuron subtypes, VIP neurons are primarily distributed in layer 2/3 of the barrel cortex [49]. VIP neurons demonstrate spatial inhibitory preferences for upper supragranular layers [50,51,52], ensuring a balanced weak inhibition throughout the entire ACx. VIP neurons may have the most specific physiological effects among the three inhibitory neuron types in the cerebral cortex. Abnormal VIP-PC circuitry may cause abnormal brain states such as depression [53], Parkinson’s disease, and epilepsy [54,55]. VIP knockout mice display altered results in memory tasks and social behavior [56]. In addition, VIP receives the most global synaptic input from the whole brain, and its priority areas are mainly in the upper supragranular layer. The superficial neurons of ACx are responsible for the information interaction between the two hemispheres of the brain. When this information connection is abnormal, it makes it hard for schizophrenia patients to finish the complex behavioral tasks, and it is also considered to be the underlying basis of generalized epilepsy syndrome.

It is currently widely believed that the imbalance between excitatory and inhibitory influences (E/I imbalance) in the brain has been proposed as a potential mechanism. GABA may play an important role in pathophysiology [1,2,3,4,5,6,7,57,58]. However, some studies have found that glutamate imbalance may have a broader impact on AVHs patients [59]. Except for the impact of gene expression differences between the human brain and mice on microcircuit function [60], the Glu–Glu imbalance could be downstream of local Glu–GABA imbalance or differential recruitment of distinct GABAergic interneuron subtypes by long-range glutamatergic inputs [61]. In addition, other subclasses of GABAergic neurons might compensate for the reduced GABA production in parvalbumin neurons, or they could be stimulated by increased glutamergic activity [62]. These results suggest that the E/I imbalance may occur on multiple levels, including the networks between brain regions and neuronal circuits within regions.

Due to the different targeting patterns of various interneuron subtypes on excitatory cells, this may affect the connectivity between different cell types, which is a limitation of current experimental methods. Therefore, here, we emphasize more on the spatial pattern of inhibition. Our study combined transgenic mice with optogenetics to selectively control specific neuron types, analyzed the spatial inhibitory characteristics of VIP neurons to PCs, and proposed that inhibitions of SOM and VIP neurons exhibit complementary spatial inhibitory preferences. This ensures the excitation–inhibition dynamic balance of the entire ACx column, contributing important theoretical information for the study of pathological brain circuits and the discovery of potential therapeutic targets.

Accumulating evidence supports the significant involvement of PV, SOM, and VIP circuit abnormalities in diseased brains. Considering the neural circuits of these neurons in both healthy and pathological conditions may help tailor specific interventions to restore dysfunctional host circuitry [63]. Additionally, optogenetics has potential application value in the treatment of targeting abnormal neural circuits [64,65,66]. Circuit-level research facilitates the discovery of potential disease targets, improving our understanding of cortical networks in the context of disease and leading to the emergence of cell type-specific therapies.

## Data Availability

The data presented in this study are available on request from the corresponding author.

## Supplementary Materials

The following supporting information can be downloaded at: https://www.mdpi.com/article/10.3390/bioengineering10050547/s1, Figure S1: Connection efficiency comparison among the three inhibitory cell types for L4 PCs; Figure S2: Connection efficiency comparison among the three inhibitory cell types for L5 PCs; Figure S3: Connection efficiency comparison among the three inhibitory cell types for L6 PCs.
