# Supplementary material for: Spatial Distribution of Inhibitory Innervations of Excitatory Pyramidal Cells by Major Interneuron Subtypes in the Auditory Cortex
Source: Bioengineering (Basel). 2023 May 1;10(5):547. doi: 10.3390/bioengineering10050547 (PMC10215958; doi:10.3390/bioengineering10050547)
Supplement: Supplementary file 1 [file bioengineering-10-00547-s001.zip › bioengineering-2361932-supplementary.pdf]

# *Supplementary Material*

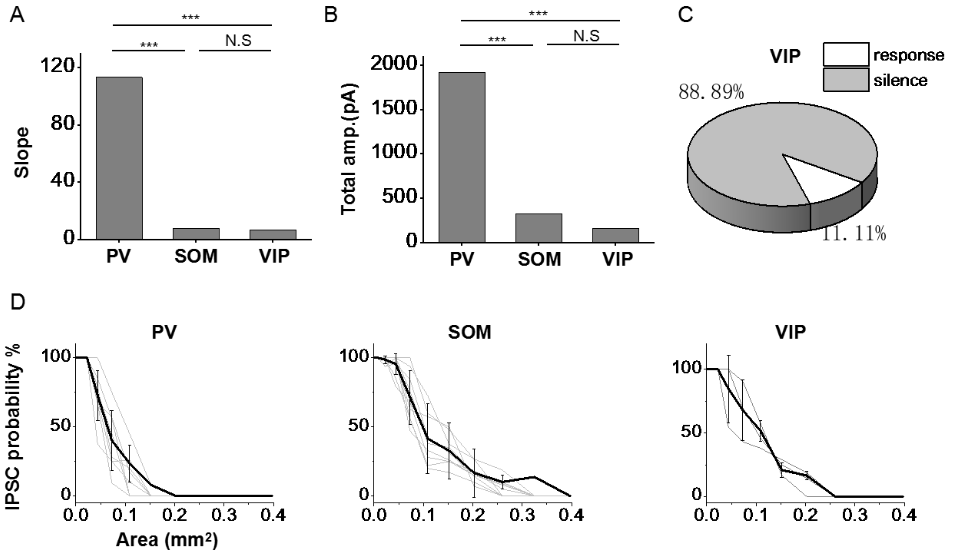


**Figure S1.** Connection efficiency comparison among the three inhibitory cell types for L4 PCs. **(A)** Slopes of the three curves in Figure 3B (PV: -113.24, SOM: -7.49, VIP: -6.43). **(B)** Comparison of total amplitude within ten laps for three interneurons (PV: 1920.25 pA, SOM: 320.52 pA, VIP: 155.35 pA). **(C)** Pie chart showing VIP connection (response N: 3/27). **(D)** IPSCs response probability in different stimulus area for three subtypes of interneurons. ***p<0.001, N.S, p>0.05.

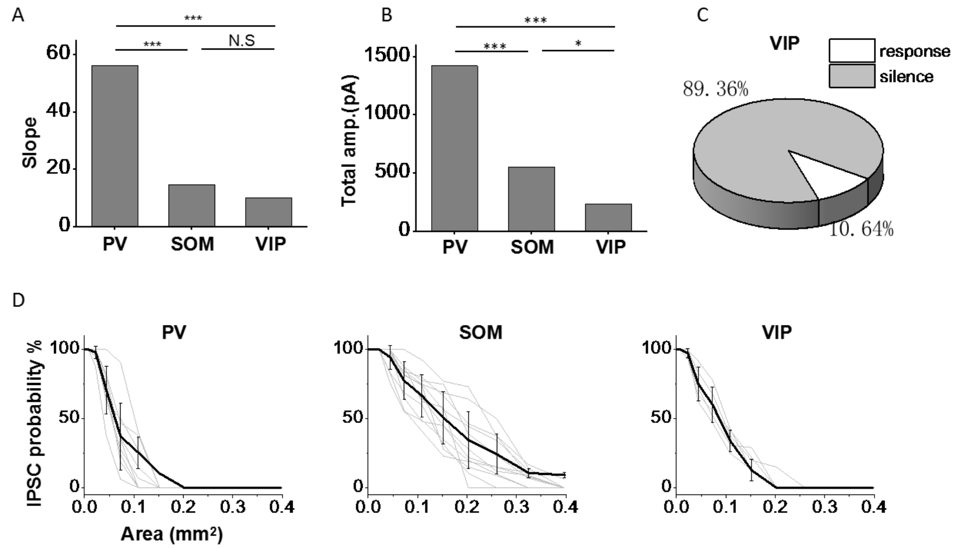


**Figure S2.** Connection efficiency comparison among the three inhibitory cell types for L5 PCs. **(A)** Slopes of the three curves in Figure 3E (PV: -56.31, SOM: -14.46, VIP: -10.01). **(B)** Comparison of total amplitude within ten laps for three interneurons (PV: 1419.78 pA, SOM: 552.28 pA, VIP: 231.30 pA). **(C)** Pie chart showing VIP connection (response N: 5/42). **(D)** IPSCs response probability in different stimulus area for three subtypes of interneurons. ***p<0.001, N.S, p>0.05.

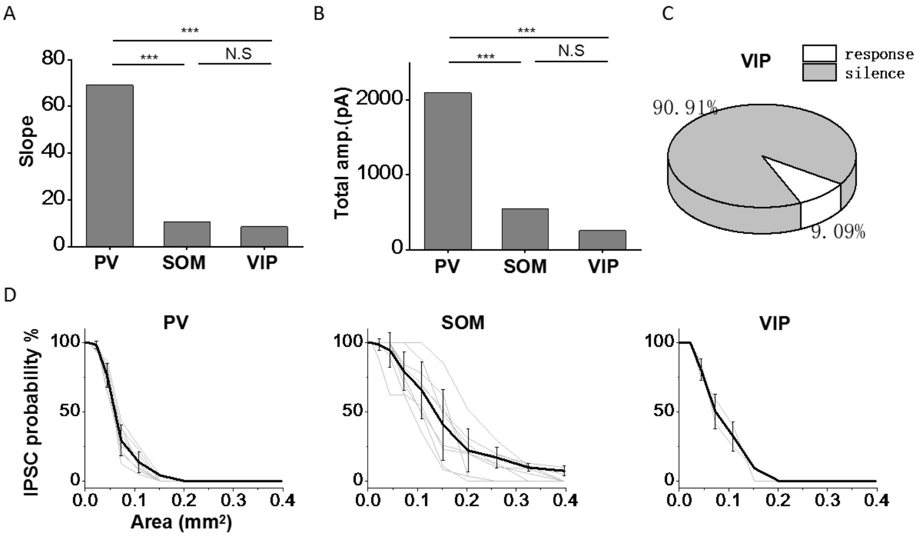


**Figure S3.** Connection efficiency comparison among the three inhibitory cell types for L6 PCs. **(A)** Slopes of the three curves in Figure 3H (PV: -69.17, SOM: -10.48, VIP: -8.47). **(B)** Comparison of total amplitude within ten laps for three interneurons (PV: 2100.88 pA, SOM: 554.72 pA, VIP: 255.48 pA). **(C)** Pie chart showing VIP connection (response N: 2/20). **(D)** IPSCs response probability in different stimulus area for three subtypes of interneurons. ***p<0.001, N.S, p>0.05.
